# Chloride intracellular channel 4 participate in the protective effect of Ginkgolide B in MPP+ injured MN9D cells: insight from proteomic analysis

**DOI:** 10.1186/s12014-020-09295-6

**Published:** 2020-09-05

**Authors:** Zili Feng, Zhibin Zhu, Wang Chen, Yu Bai, Daihua Hu, Jia Cheng

**Affiliations:** School of Bioscience and Engeering, Shaanxi University of Technology, No. 1 Donghuan 1st Road, Hanzhong, 732001 Shaanxi People’s Republic of China

**Keywords:** Ginkgolide B, GB derivatives, Parkinson’s disease, Chloride intracellular channel 4

## Abstract

**Background:**

Ginkgolide B (GB), the extract of *G. biloba* leaves, has been shown to be protective against many neurological disorders, including Parkinson’s disease (PD). Efforts have been made to synthesized ginkgolides analogs and derivatives with more targeted and smaller molecular weight. In the present study, four GB derivatives (GBHC-1-GBHC-4) were synthesized, and their protective roles in N-methyl-4-phenylpyridinium (MPP +) injured MN9D dopaminergic neuronal cell line were evaluated. Also, cell response mechanisms upon these GB derivatives treatment were analyzed by iTRAQ proteomics.

**Methods:**

MN9D cells were treated with MPP + to induce in vitro cell models of PD. Four GB derivatives (GBHC-1-GBHC-4) were synthesized, and their protective roles on cell viability and apoptosis in in vitro PD model cells were evaluated by CCK8 assay, fluorescence-activated cell sorting and DAPI staining, respectively. The proteomic profiles of MPP+ injured MN9D cells pretreated with or without GB and GB derivatives were detected using the isobaric tags for relative and absolute quantification (iTRAQ) labeling technique.

**Results:**

Pretreatment with GBHC-1-GBHC-4 noticeably increased cell viability and attenuated cell apoptosis in MPP+ -injured MN9D cells. Using proteomic analysis, we identified differentially expressed proteins upon GB and GB derivatives treatment. Chloride intracellular channel 4 (CLIC4) and “protein processing in endoplasmic reticulum” pathways participated in the protective roles of GB and GBHC-4. GB and GBHC-4 pretreatment could significantly reverse MPP+ -induced CLIC4 expression and translocation from cytoplasm to nucleus of MN9D cells.

**Conclusions:**

Quantitative comparative proteomic analysis identified differentially expressed proteins associated with GB and GB derivatives. We further verified the expression of CLIC4 by western blotting and immunocytochemistry assay. This bio-information on the identified pathways and differentially expressed proteins such as CLIC4 provide more targeted directions for the synthesis of more effective and targeted GB derivatives for the treatment of neurological disorders.

## Background

Parkinson’s disease (PD) is one of the most common neurodegenerative disorders of the central nervous system, characterized by chronic and progressive loss of midbrain dopaminergic (DA) neurons [1]. It has been known that α-Syn accumulation results in synucleinopathies that include PD, and efficient clearance of aggregated α-Syn represents a potential approach in PD therapy [2]. Current therapy strategies aim at symptomatic relief rather than preventing disease progression. Considering how rapidly population of PD grows, there is comparatively less development of conventional methods so that alternative preventions and treatments for PD has been drawing attention.

*Ginkgo biloba* is an ancient Chinese tree that has long been used for the therapy of multiple diseases [3–5]. The extract of *G. biloba* leaves have been used as effective dietary supplements and phytomedicines. Its neuroprotective effects against various cardiovascular [4] and neurological disorders, such as ischemia [6], Alzheimer’s disease [7] and depression [8] are being investigated. Researchers have prepared ginkgolides, which include Ginkgolide A (GA), Ginkgolide B (GB), Ginkgolide C (GC), Ginkgolide J (GJ), Ginkgolide K (GK), Ginkgolide L (GL) and Ginkgolide M (GM) [5]. Also efforts have been made to synthesized ginkgolides analogs and derivatives. GB cinnamate, a synthesized GB analog, had increased blood brain barrier (BBB) permeation compared to GB, with a 1.61-fold increase in half-life of SD rats [9]. Pre-administration with XQ-1H, a derivate of GB, suppressed hyperlipidemia, reduced cerebral infarct size, improved BBB permeability and diminished brain edema after a stroke in hyperlipidemic rats [10, 11]. In vitro study showed GB and bilobalide have a protective effect on apoptosis induced by α-Syn aggregate, and an enhancement on clearance of α-Syn by astrocytic, which gives us an insight into prevention of PD with effective dietary supplements in future [12, 13].

Our lab is making an effort to synthesized small molecule drugs of GB derivatives with more targeted effects. Therefore, it was of interest to elucidate the cell response mechanisms of these GB derivatives, to provide more directions in the synthesize of small molecule drugs. In this study, the neuroprotective effects of GB derivatives (GBHC-1, GBHC-2, GBHC-3, GBHC-4) in a PD cell model induced by MPP+ were detected, and the cell response mechanisms upon GB derivatives pretreatment were also investigated using iTRAQ-labeled proteome analysis.

## Methods

### Cell culture

The dopaminergic neuronal cell line MN9D (American Type Culture Collection, Manassas, VA, USA) was cultured in high-glucose Dulbecco’s modified Eagle’s medium (DMEM) (Gibco Laboratories, Grand Island, NY, USA) containing 10% fetal bovine serum, penicillin (100 units/mL), and streptomycin, and incubated at 37 °C in a humidified atmosphere with 5% CO_2_. The culture media was replaced every 2 to 3 days. MN9D cells were treated with 100 μmol/L MPP+ (Sigma-Aldrich Chemical Company, St Louis, MO, USA) for 24 h to induce in vitro cell models of PD for subsequent experiments. Cells were grouped as follows: control group (MN9D cells), PD group (MN9D cells treated with 100 μmol/L MPP+), GB group (MN9D cells were pretreated with GB, and then cells were incubated with 100 μmol/L MPP+), GBHC1-GBHC4 groups (MN9D cells were pretreated with GBHC1-GBHC4, and then cells were incubated with 100 μmol/L MPP+). Cells were harvested for western blotting, RT-PCR or proteomics analysis. For cell treatment, GB and GB derivatives were dissolved in DMSO, and were diluted with complete medium to a final concentration of 5, 10, 25, 50, 75,100 µM. The optical concentration of GB and GB derivatives were determined according to their effect on cell viability and apoptosis.

### Cell viability determination

The Cell Counting Kit (Cat#CK04-01, Dojindo, Kumamoto, Japan) was used according to the protocol. Briefly, after MPP + exposure, cells in 100 μL culture medium in the 96-well plate were incubated with 10 μL CCK-8 solution at 37 °C for 1 h. Then absorbance values at 450 nm were measured.

### DAPI staining

MN9D cells (4 × 10^5^/ml) were seeded on coverslips and treated as described above. After treatment, coverslips were washed with PBS for twice and fixed in 4% paraformaldehyde for 30 min at room temperature. Then MN9D cells on coverslips were permeabilized with 0.1% Triton X-100 and stained with 2 µg/ml DAPI for 10 min at room temperature. DAPI stained MN9D cells were observed under a Confocal Laser Scanning Microscopy (Leica, Heidelberg, Germany).

### Protein digestion, labeling and chromatography separation

Different groups of MN9D cells cultured in 100-mm^2^ dishes were washed three times with ice-cold phosphate-buffered saline (PBS). Then cells were collected and suspended with homogenization buffer (100 mM phosphate buffer, pH7.5, 10 mM KCl, 1 mM MgCl_2_, 1 mM EDTA, 10% sucrose, 1% phenylmethylsulfonyl fluoride (PMSF), 0.2% TritonX-100) containing 1% protease and 1% phosphatase inhibitor cocktails (Roche Applied Science). Each group of cells from three different 100-mm^2^ dishes were collected and mixed as one protein sample. Three protein samples of each group were collected independently. The collected cell suspension samples (three independent samples for each group) were then homogenized by sonication (1 s on, 3 s off; 30 cycles) on ice. The obtained homogenate were centrifuged at 4 °C, 15000 rpm for 15 min and the protein contents of supernatant were quantified using a BCA kit (Sigma, St.Louis, MO, USA). Protein samples were then proteolyzed with sequencing-grade trypsin and labeled with a corresponding iTRAQ label (iTRAQ Reagent-8plex multiplex kit) at room temperature for 2 h according to the manufacturer’s instructions. Excess iTRAQ reagents were removed from the pooled samples, and strong cation exchange (SCX) fractionation was performed as described previously [14].

### Protein identification, quantification and bioinformatic analysis

One microgram of peptides for each fraction were separated and characterized by Easy-nLC 1000 HPLC system coupled with Orbitrap Fusion Lumos Mass Spectrometer (Thermo Fisher Scientific, San Jose, CA, USA). Proteome Discoverer 2.1 (Thermo Fisher Scientific) was used to analyze the raw data. Mascot 2.3 (Matrix Science) embedded in Proteome Discoverer was used to search the raw data against the uniprot-human database (20190102; 177661 sequences). The peptides with a false discovery rate (FDR) less than 0.01% were used for further analysis. Fold change (FC) in protein abundance > 1.2 and a p-value ≤ 0.05 for student’s t-test were considered as having statistically significant differences. Blast 2GO program (Version 2.7.2) was used for function annotation of the differentially expressed proteins [15]. Pathways associated with the differentially expressed proteins were annotated by using the online tool KEGG Pathway (https://www.genome.jp/kegg/ pathway.html). Perseus V1.4.1.3 was used for statistical and hierarchical clustering analyses.

### Immunocytochemistry assay

CLIC4 localization was detected by immunofluorescence assay. In brief, MN9D cells of different groups cultured in confocal dishes (Corning Life Sciences) were fixed with 4% paraformaldehyde for 15 min at room temperature, followed by permeabilization with 0.3% Triton X-100 treatment for 10 min. Cells were then blocked with 3% BSA for 1 h at 37 °C and stained with primary antibody (anti-CLIC4, 1:200, Abcam, Cat#ab183043) overnight at 4 °C. After washing three times, cells were incubated with secondary antibody conjugated to Alexa Fluor-488 (1:500, Invitrogen, Carlsbad, CA, USA) for 1 h and DAPI (Invitrogen) for 20 min at 37 °C. Fluorescence images were acquired using Confocal Laser Scanning Microscopy (Leica, Heidelberg, Germany).

### Cell apoptosis by fluorescence-activated cell sorting

Flow cytometric analysis was performed using a Dead Cell Apoptosis kit with Annexin-V/FITC and propidium iodide (PI) following manufacturer’s instruction (Cat#40305ES60, Yeasen Co, Shanghai, China). Briefly, 5 × 10^5^ MN9D cells/well were seeded in 6-well plates and treated as described above. After washing in cold PBS, cells were collected and re-suspended in 1 × Annexin binding buffer at a density of 1 × 10^6^ cells/mL. Then, 5 µL of Annexin-V/FITC and 1 µL 100 µg/mL PI were added to each 100 µ L of cell suspension, followed by incubation of the samples at room temperature for 15 min in dark condition. After incubation, apoptosis was analyzed by fluorescence-activated cell sorting (FACS) using BD FACSDiva Software v6.1.3 (BD, Franklin Lakes, NJ, USA), within 1 h. Cells undergoing early and late apoptosis were Annexin-V FITC-positive.

### Western blot analysis

Different groups of MN9D cells were washed with ice cold PBS and lysed with RIPA lysis buffer (1% NP40, 0.1% SDS, 50 mM DTT) containing protease inhibitor cocktail on ice. Protein concentrations in the supernatants were measured using a BCA kit. For the detection of cytoplasmic and nuclear resident CLIC4, cytoplasmic and nuclear protein lysates of MN9D cells were extracted using ExKine™ Nuclear and Cytoplasmic Protein Extraction kit (Abbkine, Cat: KTP3001) according to the manufacturer’s instruction. Protein lysates were resolved using 10% SDS-PAGE and then transferred to polyvinylidene difluoride membranes (PVDF, Millipore, Merck KGaA, Darmstadt, Germany). The membranes were blocked with 5% non-fat milk at 37 °C for 1 h. The blocked membranes were washed with 1 × TBST for three times, 5 min for each time. Then membranes were then incubated with specific primary antibodies overnight at 4 °C(Anti-β-actin, Cat#ab179467, 1:10000 or anti-CLIC4 antibody, Cat#ab183043, 1:8000 or anti-Histone H3, Cat#4499, 1:2000 or anti-α-Tubulin, Cat#2125, 1:5000). After washing, the membranes were incubated with secondary antibodies (Invitrogen, Cat#31460, 1:200000) at room temperature for 1 h. Incubated membranes were then washed and visualized using PierceTM ECL Plus Western Blotting Substrate (Thermo Fisher Scientific, Inc., Waltham, MA, USA). Image Studio Lite software (version 4.0) was used for quantification. Experiments were repeated at least three times.

### Quantitative RT-PCR

Total RNA from MN9D cells was extracted using TRIzol reagent (Invitrogen Life Technologies) according to the protocol of the manufacturer. A TransScript First-strand cDNA Synthesis SuperMix kit was used to cDNA synthesis. Quantitative RT-PCR was performed using a SYBR Premix Ex Taq kit (TaKaRa, Japan). Targeted gene expression was calculated using the 2^−ΔΔCt^ method while β-actin was used as internal control.

### Statistical analysis

All experiments were performed at least three times independently. Data presented in a graphical format were performed using GraphPad Prism 7.0 (GraphPad, SanDiego, CA, USA) and expressed as the mean ± SD. Comparison between two independent samples was performed using a Student’s t test. One-way analysis of variance (ANOVA) followed by a Fisher’s least significant difference test was applied for comparisons between multiple groups. The statistical significance was set at p < 0.05.

## Results

### Protective effects of ginkgolides and ginkgolide derivates against MPP+ induced toxicity in MN9D cells

**Fig. 1:**
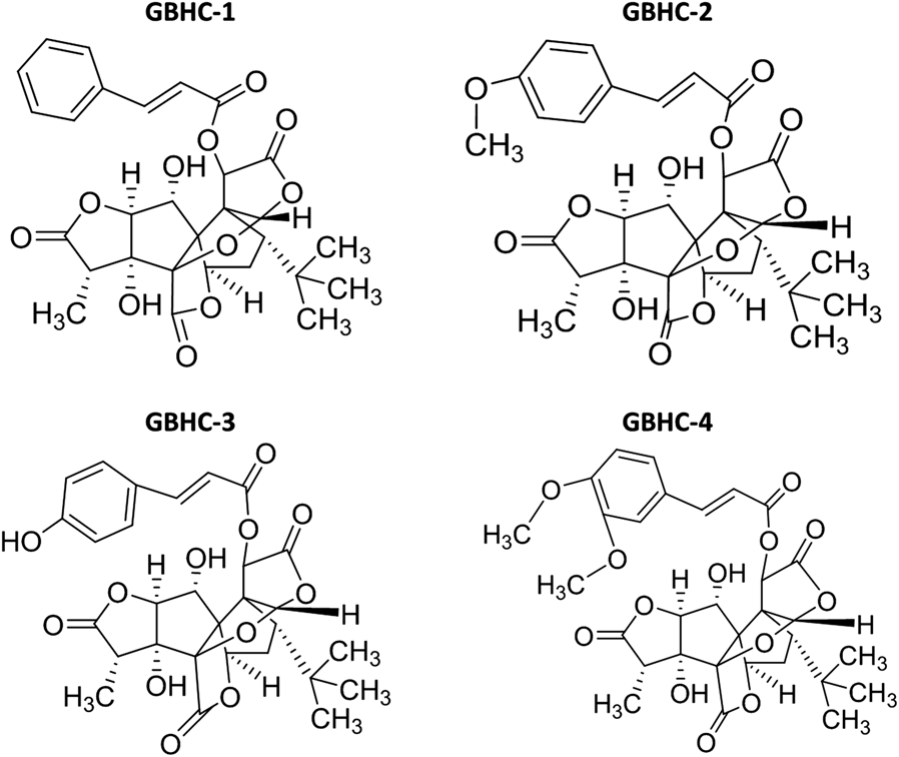
The structures of four derivatives of Ginkgolide B

**Fig. 2:**
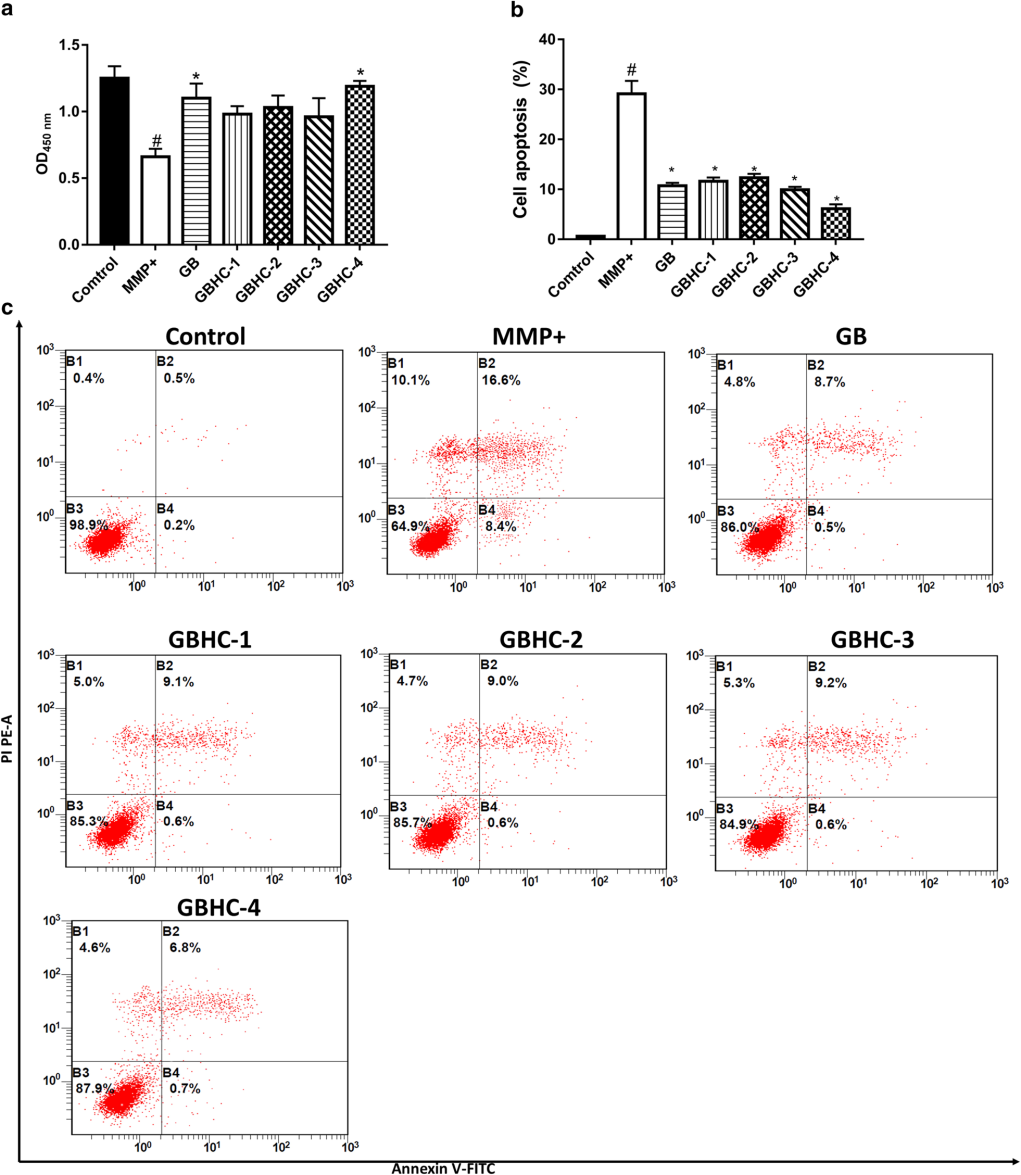
Ginkgolides and ginkgolide derivates treatment increase cell viability (**a**) and inhibit MPP+ induced cell apoptosis (**b** and **c**). # compared with control group, P < 0.05; *compared with MPP+ group, P < 0.05

**Fig. 3:**
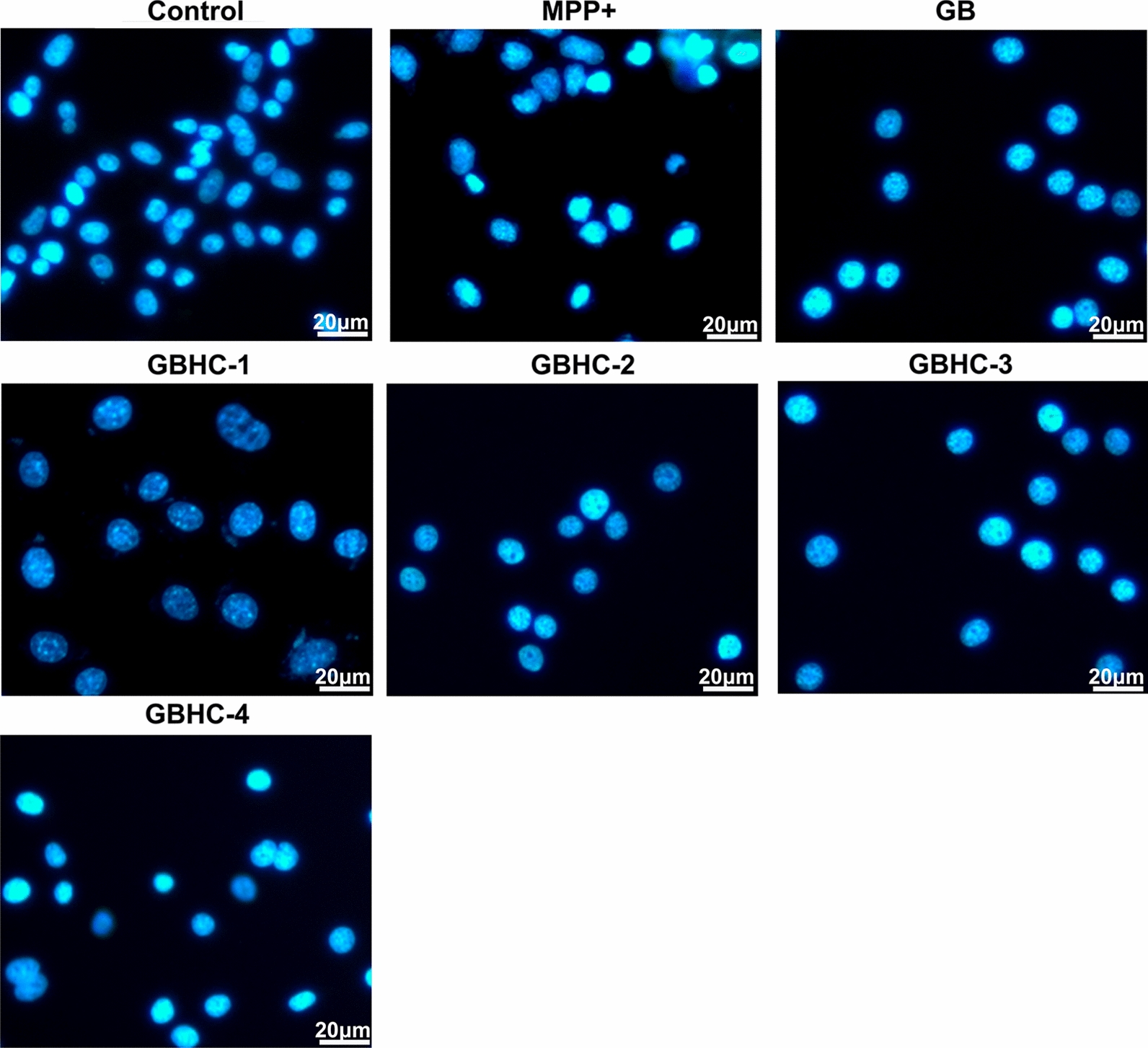
The morphological changes of apoptosis bodies in MPP+ injured MN9D cells with or without GB or GB derivatives pretreatment. Bar = 20 µm

In this study, four derivatives of Ginkgolide B were synthesized in our lab, and the structures were shown in Fig. 1. We first compared the neuro-protective effect of GB and GB derivatives. MN9D cells were pretreated with or without GB and GB derivatives (5, 10, 25, 50, 75,100 µM) prior to MPP+ injury. MPP+ treatment could significantly reduce cell viability and induce cell apoptosis compared with control group (P < 0.05). While pretreatment with different concentrations of GB and GB derivatives (5-50 µM) increase MN9D cell viability and decreased cell apoptosis in a dose dependent manner. 70 µM and 100 µM of GB and GB derivatives pretreatment slightly aggravate the injury of MPP+ on MN9D cell (Additional file 1). For further evaluation, 50 µM of GB and GB derivatives were selected. Pretreatment with GB and GB derivatives at a concentration of 50 μM increase MN9D cell viability in varying degree. Compared with MPP+ alone group, pretreated with GB and GBHC-4 both significantly increased cell viability (Fig. 2a P < 0.05) and alleviate MPP + induced MN9D cell apoptosis (Fig. 2b and c, P < 0.05). The morphological changes were also observed using DAPI staining. The number of apoptotic bodies in the MPP+ treated groups were markedly increased compared with the control group, and nuclear membrane broken were observed (Fig. 3). Pretreated with GB and GBHC-4 both could significantly reduce the number of apoptotic bodies in MPP+ injured MN9D cells. These results suggested that GB derivatives, especially GBHC-4 could recover cell viability and inhibit apoptosis against MPP+ toxicity.

### Proteomic analysis

In order to investigate the cellular response mechanism of GB and GB derivatives, and provide more improvement directions for further synthesis, the total proteins of MN9D cell pretreated with GB and GBHC-4 were collected for proteome analysis. The quantitative proteomics experiments resulted in the identification of 1143 proteins. Differential expressed proteins (DEPs) were identified using pairwise comparison of protein abundance. As to criteria of significance, both a cut off 1.2 for protein fold change and a statistical significance threshold of p < 0.05 were applied. Compared with control group, 65 DEPs were identified in MPP+ injured PD group, with 29 ones were significantly upregulated and 36 ones were significantly downregulated (Additional file 2). In GB pretreatment group, 103 DEPs were identified compared with MPP+ group, with 39 ones were upregulated and 64 ones were downregulated (Additional file 3). While in GBHC-4 group, 96 DEPs were identified compared with MPP+ group, with 39 ones were upregulated and 57 ones were downregulated (Additional file 4).

GO terms were assigned to the DEPs to explore their functions and biological processes (Additional file 5) and KEGG pathway mapping was conducted to better identify the potential involvement of these DEPs in cellular biological pathways (Additional file 6). In order to clarify the overlap of these DEPs from different groups, a Venn diagram (Additional file 7) was constructed. In MPP+ group, pathways related to animo acid metabolism were enriched. Upon GB pretreatment, pathways related to phagosome, pathogenic *E. coli* infection and protein in endoplasmic reticulum were enriched, while GBHC-4 pretreatment enriched pathway associated with arginine and protein metabolism, aminoacyl-tRNA biosynthesis, Fc γ R-mediated phagocytosis and endocytosis. Among all the DEPs and the involved biological pathways, we focus on the DEPs with opposite tendency between MPP+ injured PD group and GB or GBHC-4 pretreated PD groups. As a result, a chloride intracellular channel protein CLIC4 were screened out. Compared with control MN9D cells, the expression of CLIC4 in MPP+ injured cells was significantly increased by 2.56 folds (P = 0.036, Additional file 2). While GB (P = 0.007) and GBHC-4 (P < 0.000) pretreatment could significantly reduce its expression compared with MPP+ injured group (Additional files 3 and 4). Most of the DEGs enriched pathways were related to cell metabolism (Additional file 5). Among all these pathways, CLIC4 related pathway “protein processing in endoplasmic reticulum”, may participate in the protective role of GB and GBHC-4.

### MPP+ injured MN9D cells showed increased total expression of CLIC4

**Fig. 4:**
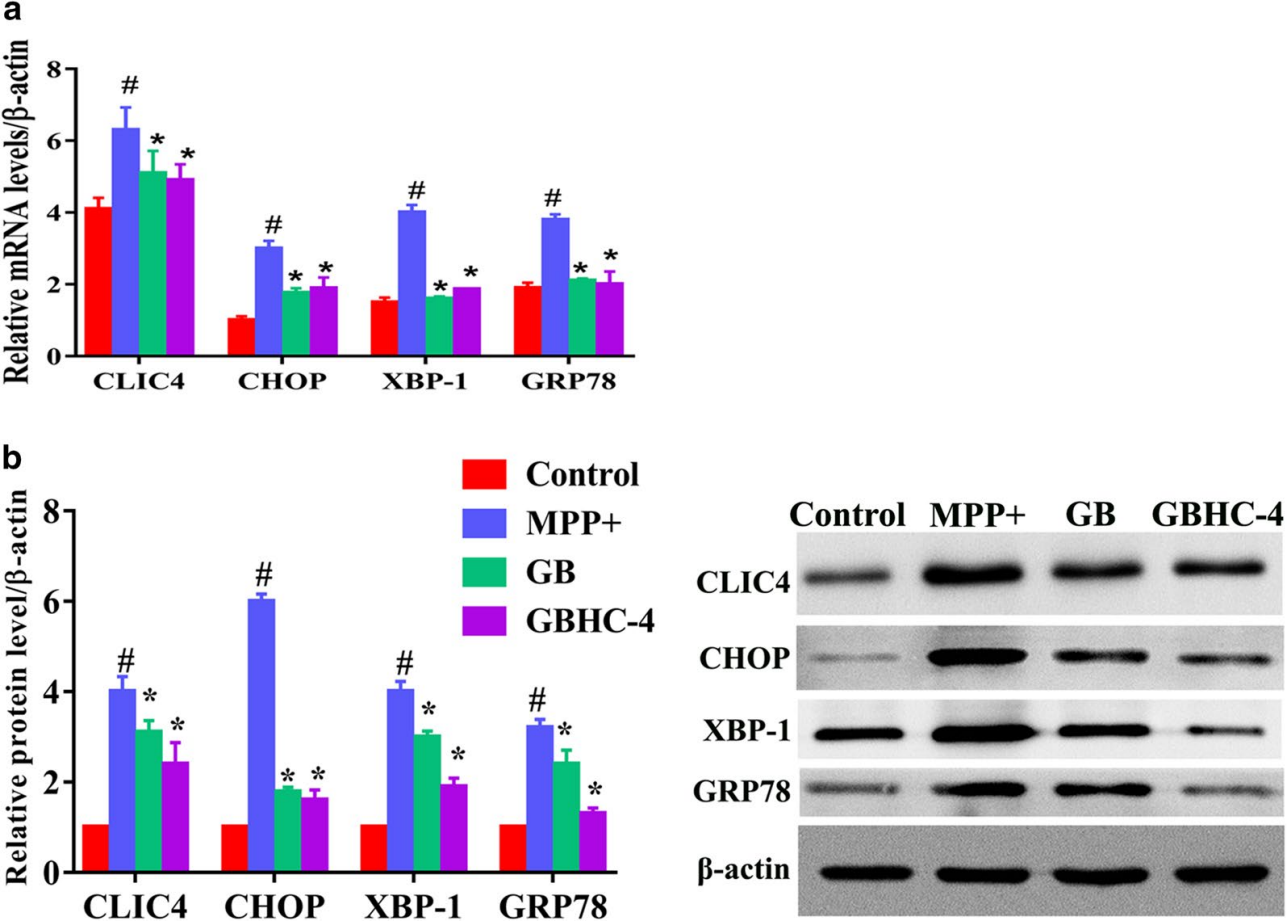
mRNA and proteins expression changes of CLIC4 and ER-related apoptotic proteins in MPP+ injured MN9D cells. **a** mRNA expression changes, **b** protein expression changes. # compared with control group, P < 0.05; *compared with MPP+ group, P < 0.05

Among all these DEPs enriched pathways, CLIC4 and its related pathway “protein processing in endoplasmic reticulum” were intriguing. The proteome results showed that the expression of CLIC4 were significantly increased in PD model cells injured by MPP+. We further verified the expression change of CLIC4 and endoplasmic reticulum (ER)-related apoptosis proteins using qPCR and western blotting. As shown in Fig. 4a, compared with normal MN9D cells, MPP+ toxicity significantly increased the mRNA level of CLIC4 and ER-related apoptosis proteins (P < 0.05). GB and GBHC-4 pretreatment could significantly decrease MPP+ induced upregulation of these mRNA (P < 0.05). The protein expression showed consistent tendency (Fig. 4b).

### GB pretreatment inhibit subcellular translocation of CLIC4

**Fig. 5:**
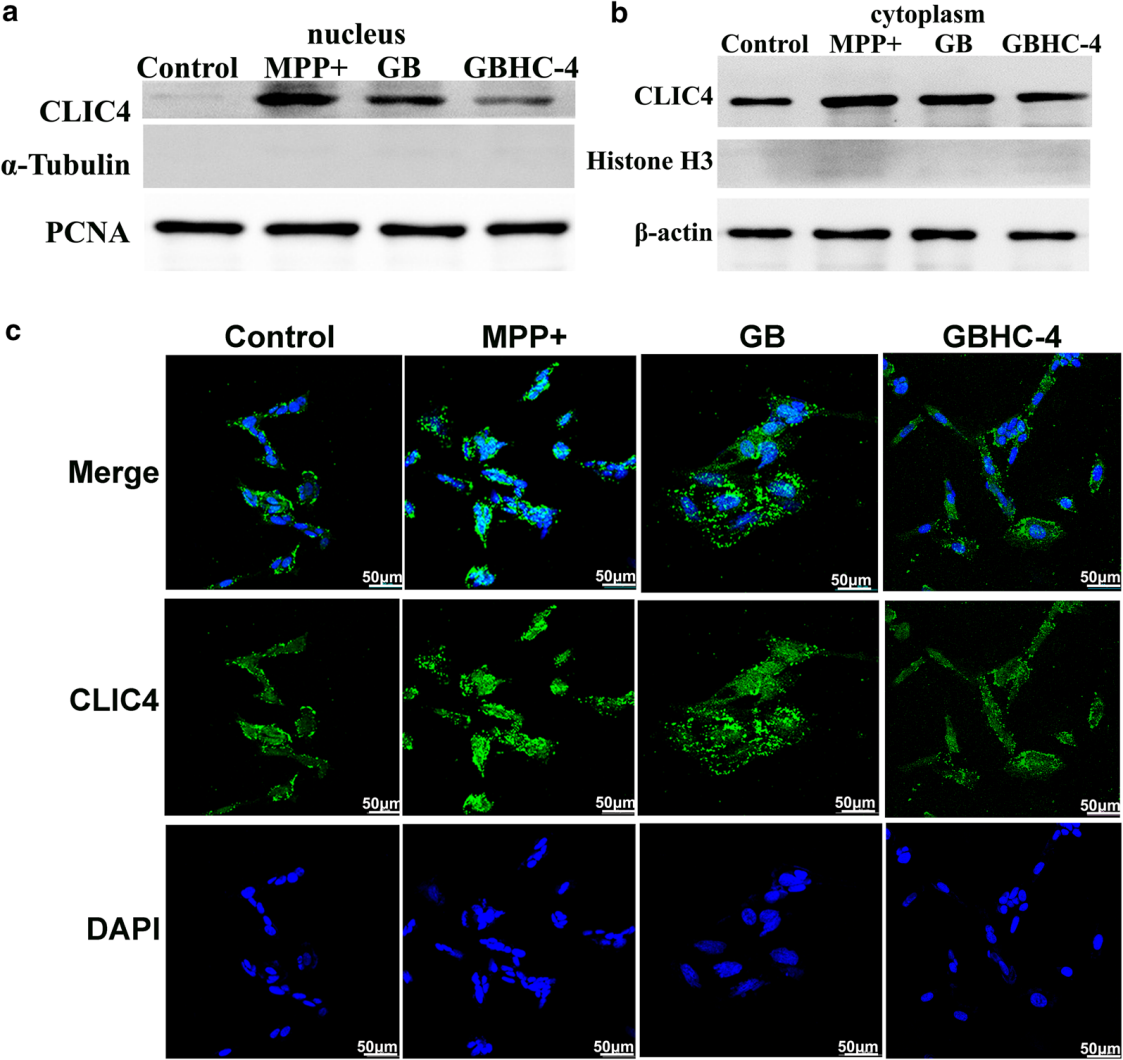
Subcellular expression and translocation of CLIC4 in MPP + injured MN9D cells with or without GB or GBHC-4 pretreatment. **a** Nuclear and **b** cytoplasmic expression of CLIC4 expression in different groups were detected using western blotting; **c** Subcellular expression and translocation of CLIC4 were detected by immunofluorescence, Bar = 50 µm

It has been reported that subcellular localization of CLIC4 is associated with its pro-apoptotic and differentiation functions, and translocation of CLIC4 from cytoplasm to the nucleus of cells were found in cells undergoing a variety of stress responses. We further extracted cytoplasmic and nuclear proteins to determine the subcellular level change of CLIC4. Compared with normal MN9D cells, MPP+ toxicity significant increased both cytoplasmic and nuclear residence of CLIC4 (Fig. 5a and b). However, the expression and intracellular trafficking of CLIC4 from cytoplasm to the nucleus upon MPP+ injure are dynamic and reversible. GB pretreatment could significantly counteract MPP+ induced upregulation of CLIC4, both in cytoplasm and nuclear (Fig. 5a). In normal MN9D cells, CLIC4 located mainly in cytoplasm or around the nucleus. Upon MPP+ injure, increased CLIC4 translocated from cytoplasm to the nucleus, while GB or GHBHC-4 pretreatment could significantly reverse MPP+ induced translocation of CLIC4 (Fig. 5c).

## Discussion

Knowledge about specific changed proteins or molecules may allow the determination of specific cellular response, and help us to understand the physiological and pathological processes. In this study, we have characterized the proteome changes and associated cellular response of GB and GB derivatives in MPP+ injured MN9D cells. Currently, multiple reports on the neuroprotective properties of GB exist [16, 17]; however, there is less reported evidence regarding the specific targets of GB. Our lab has been made our effort to synthesis various GB derivatives to select more targeted and small-molecule ones. The findings in this study presented knowledge about the changed proteins and associated cellular response, which would help us to understand the mechanism and potential targets by which GB and GB derivatives modulate neural and cognitive functions.

In the present study, we first examined the effect of GB and GB derivatives on MPP+ injured MN9D cell death in vitro, and the protective role of GB were consistent with previous reports. Also, GBHC-4 (its structure is shown in Fig. 1) showed promoted protection than GB and other derivatives. We demonstrated 103 DEPs in GB pretreatment group compared with MPP+ injured PD group, with 40 upregulated ones and 63 downregulated ones. While in GBHC-4 group, 96 DEPs were identified compared with PD group, with 40 upregulated ones and 56 downregulated ones. KEGG results suggested that the most enriched KEGG pathways of DEPs in PD group (compared with control group) were arginine and proline metabolism, selenocompound metabolism, tryptophan metabolism and cysteine and methionine metabolism. The enrichment pathways of alanine, aspartate, glutamate, and purine metabolism might act as alternative pathways to overcome inadequate glucose supply and energy crisis in neurodegeneration as previously reported [18]. Upon GB pretreatment, pathways related to phagosome, pathogenic *E. coli* infection and protein in endoplasmic reticulum were enriched, while GBHC-4 pretreatment enriched pathway associated with arginine and protein metabolism, aminoacyl-tRNA biosynthesis, Fc γ R-mediated phagocytosis and endocytosis. There are reported evidence supporting that phagosomes fuse with the ER. Phagocytosis is a key cellular pathway of innate immune response, and it is critical for both defending against invading pathogens and maintaining tissue homeostasis. The pathological hallmark of PD is the presence of α-synuclein (α-syn)-rich Lewy bodies, and an altered phagocytic function of microglia in PD have been described in several studies [19–21]. In vivo administration of GB and GB derivatives may exert roles on microglial phagocytic function, which needs further verifications. Here, we found that phagocytosis and proteins in ER pathways are enriched, suggesting that in addition to the protective role on cell viability and cell death, GB may regulate critical homeostatic mechanisms of MN9D cells. Further studies regarding these processes should be performed to help us to understand the protective mechanisms of GB.

In GB and GBHC-4 groups, several proteins were found to be involved in more than one pathway. We found Chloride intracellular channel 4 (CLIC4) play important roles in “protein processing in endoplasmic reticulum”. The CLIC family of chloride intracellular channels is composed of seven differentially expressed proteins that localize to the cytoplasm and intracellular organelles in many cell types [22, 23]. CLIC1- CLIC5 are highly homologous in size and sequence, whereas the larger CLIC5B and CLIC6 have extended amino-terminal domains [24, 25]. CLICs are present in neurons and astrocytes, and they are reported to play critical roles in neuro-physiological and pathological conditions, such as Alzheimer’s disease [26]. CLIC1 is required for α-amyloid-induced generation of reactive oxygen species by microglia and participate in microglia-mediated α-amyloid-induced neurotoxicity [27–29]. CLIC2 mutation was identified on Xq28 in a male with X-linked intellectual disability (XLID) [30]. However, in this study, only CLIC4 were identified as differentially expressed proteins in MPP + and GB/GBHC-4 treated MN9D cells (fold change > 1.5 and P < 0.05). CLIC4 exists in both soluble and membrane bound forms and is structurally related to the omega class of glutathione transferases [31, 32]. Our study for the first time reported that CLIC4 might be a potential target of GB and GBHC-4. Upon 6-OHDA toxicity, the expression of CLIC4 were significantly increased, which was consistent with previously reports that CLIC4 was accumulated in apoptotic neurons [33]. Interesting, we also found that increased CLIC4 translocated from cytoplasm to nuclear, while GB and GBHC-4 pretreatment could significantly decrease CLIC4 expression and translocation to nuclear. Nuclear translocation of CLIC4 as a coincident event in GB’s protective role was quite inspiring in this study. However, the functions of nuclear CLIC4 and the physiological translocation of CLIC4 in MN9D cells have not been clarified. Studies have shown that CLIC4 binds to many other molecule, such as actin, tubulin and 14-3-3 isoforms, suggesting that CLIC4 has broader molecular functions [31, 34]. Further research on the functions of CLIC4 in GB and GBHC-4 protective role and its interacting proteins may provide us with more guidelines for the synthesis of targeted GB derivatives.

In this study, only undifferentiated MN9D cells injured by MPP+ were used to evaluate cell loss and viability while α-synuclein accumulation was not detected due to the limitations of the MN9D cell model. This PD cellular models did not represent all aspects of PD. Further in vitro and in vivo studies are needed to verify all aspects of PD changes, including the loss of dopaminergic neurons in the substantia nigra pars compacta and the develop of a-synuclein aggregates.

## Conclusions

We have demonstrated the proteome profile of MN9D cells upon GB and GB derivatives protection against MPP+ injure by iTRAQ proteomics. The overall data analysis revealed differentially expressed proteins, and proteins associated with the protective roles of GB derivatives in MPP+ -induced MN9D cells. However, further studies are needed to investigate the functions and effects of these changed proteins to better understand the protective role of GB in PD. Also the results will shed lights on the targets of GB’s protecting roles, which will help us to synthesized more targeted and effective small molecule drugs of GB derivatives.

## Supplementary information

**Additional file 1:** The changes of MN9D cell viability upon pretreatment of different concentration of (5–50 µM) GB and GB derivates.

**Additional file 2:** Differential expressed proteins (DEPs)in MPP+ group compared with control group.

**Additional file 3:** Differential expressed proteins (DEPs)in GB group compared with MPP+ group.

**Additional file 4:** Differential expressed proteins (DEPs)in GBHC-4 group compared with MPP+ group.

**Additional file 5:** DEPs assigned GO terms. A: GO terms assigned to DEPs in MPP+ group compared with control. B: GO terms assigned to DEPs in GB group compared with MPP+ group. C: B: GO terms assigned to DEPs in GBHC-4 group compared with MPP+ group.

**Additional file 6:** DEPs assigned KEGG pathways. A: KEGG pathways associated with DEPs in MPP+ group compared with control. B: KEGG pathways associated with DEPs in GB group compared with MPP+ group. C: B: KEGG pathways associated with DEPs in GBHC-4 group compared with MPP+ group.

**Additional file 7:** Venn diagram showed the common DEPs in different groups.

## Data Availability

The datasets used and/or analyzed during the current study are available from the corresponding author on reasonable request.

## Abbreviations

GB: Ginkgolide B
PD: Parkinson’s disease
CLIC4: Chloride intracellular channel 4
KEGG: Kyoto Encyclopedia of Genes and Genomes
GO: Gene ontology
