# Supplementary figures and images for: Chloride intracellular channel 4 participate in the protective effect of Ginkgolide B in MPP+ injured MN9D cells: insight from proteomic analysis
Source: Clin Proteomics. 2020 Sep 5;17:32. doi: 10.1186/s12014-020-09295-6 (PMC7487930; doi:10.1186/s12014-020-09295-6)

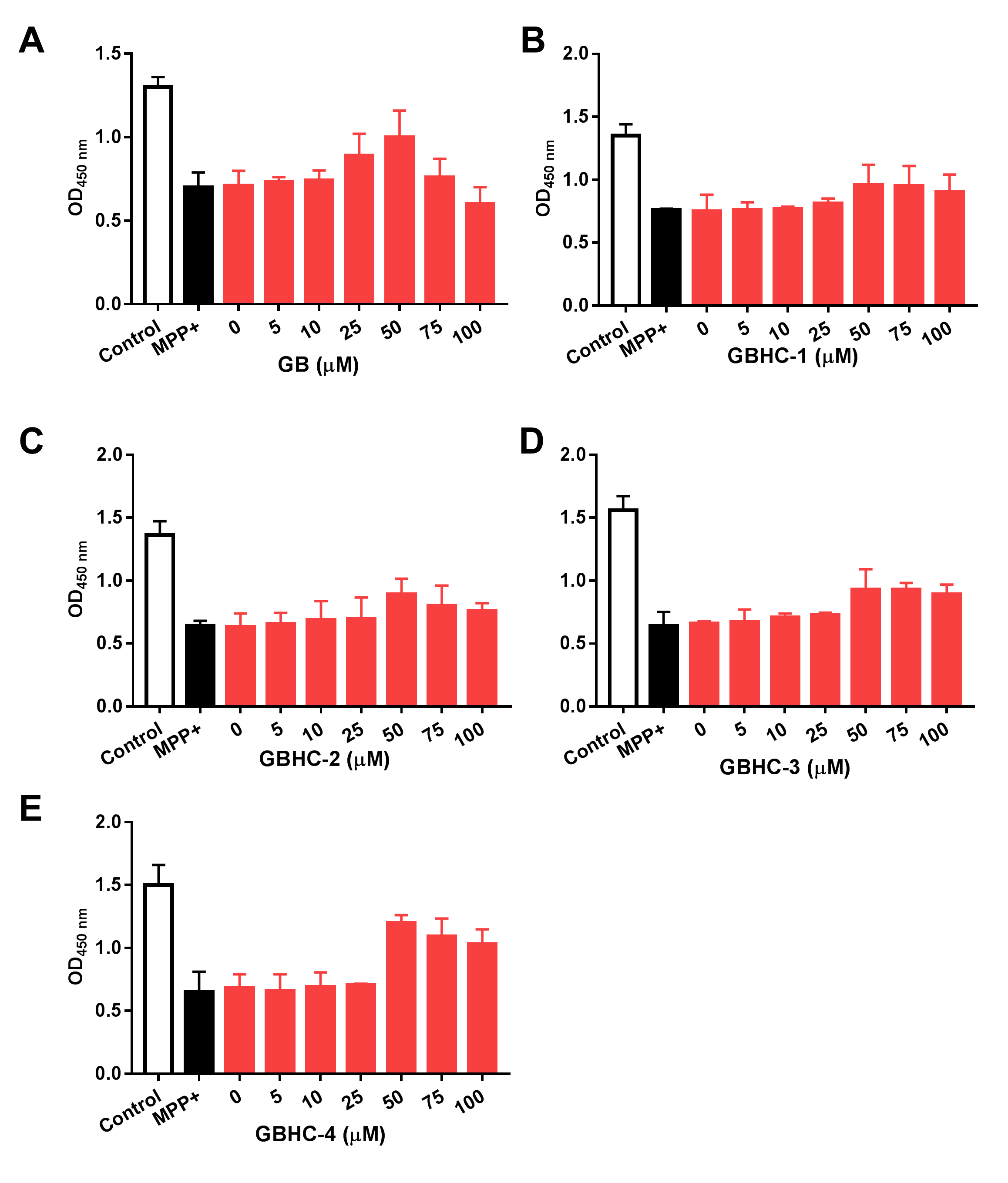

Supplement: Supplementary file 1 — Additional file 1: The changes of MN9D cell viability upon pretreatment of different concentration of (5–50 µM) GB and GB derivates. [file 12014_2020_9295_MOESM1_ESM.tif]

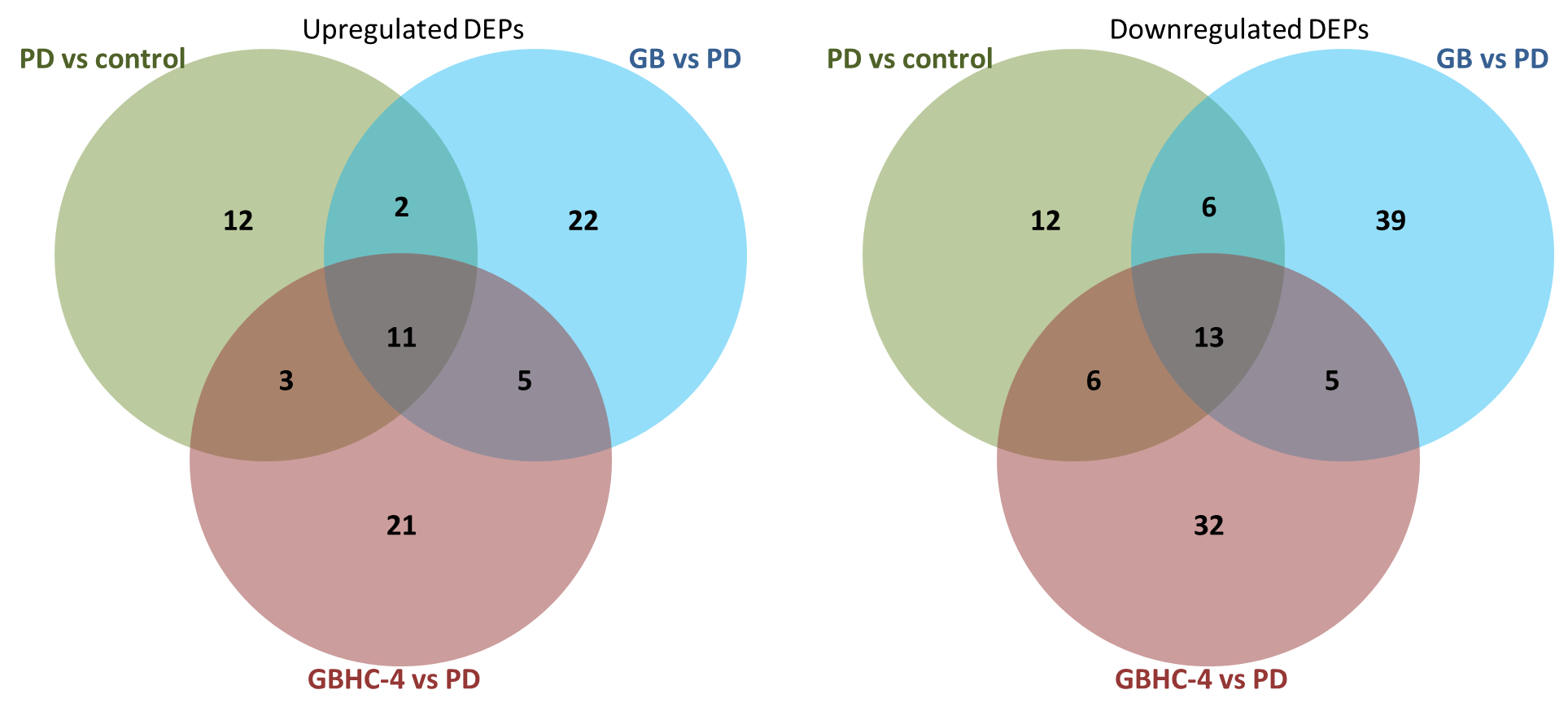

Supplement: Supplementary file 7 — Additional file 7: Venn diagram showed the common DEPs in different groups. [file 12014_2020_9295_MOESM7_ESM.tif]
